# Supplementary material for: Polygenic and socioeconomic risk for high body mass index: 69 years of follow-up across life
Source: PLoS Genet. 2022 Jul 14;18(7):e1010233. doi: 10.1371/journal.pgen.1010233 (PMC9282556; doi:10.1371/journal.pgen.1010233)
Supplement: S1 STROBE Checklist — (DOC) [file pgen.1010233.s001.doc]

STROBE Statement—Checklist of items that should be included in reports of ***cohort studies***

|  | Item No | Recommendation | Location in paper |
| --- | --- | --- | --- |
| **Title and abstract** | 1 | (*a*) Indicate the study’s design with a commonly used term in the title or the abstract | Title, abstract |
|  |  | (*b*) Provide in the abstract an informative and balanced summary of what was done and what was found | Abstract |
| Introduction | | |  |
| Background/rationale | 2 | Explain the scientific background and rationale for the investigation being reported | Introduction paragraphs 1-6 |
| Objectives | 3 | State specific objectives, including any prespecified hypotheses | Introduction paragraph 6 |
| Methods | | |  |
| Study design | 4 | Present key elements of study design early in the paper | Methods, paragraph 1 |
| Setting | 5 | Describe the setting, locations, and relevant dates, including periods of recruitment, exposure, follow-up, and data collection | Methods, paragraph 1 |
| Participants | 6 | (*a*) Give the eligibility criteria, and the sources and methods of selection of participants. Describe methods of follow-up | Methods, paragraph 1 |
|  |  | (*b*) For matched studies, give matching criteria and number of exposed and unexposed | n/a |
| Variables | 7 | Clearly define all outcomes, exposures, predictors, potential confounders, and effect modifiers. Give diagnostic criteria, if applicable | Methods, measures section |
| Data sources/ measurement | 8* | For each variable of interest, give sources of data and details of methods of assessment (measurement). Describe comparability of assessment methods if there is more than one group | Methods, measures section |
| Bias | 9 | Describe any efforts to address potential sources of bias | Methods, Statistical analysis and Sensitivity analyses section |
| Study size | 10 | Explain how the study size was arrived at | Methods, Sensitivity analyses section paragraph 2 |
| Quantitative variables | 11 | Explain how quantitative variables were handled in the analyses. If applicable, describe which groupings were chosen and why | Methods, Statistical analysis section |
| Statistical methods | 12 | (*a*) Describe all statistical methods, including those used to control for confounding | Methods, Statistical analysis section |
|  |  | (*b*) Describe any methods used to examine subgroups and interactions | Methods, Statistical analysis section |
|  |  | (*c*) Explain how missing data were addressed | Methods, statistical analyses paragraph 4 and Sensitivity analyses section paragraph 2 |
|  |  | (*d*) If applicable, explain how loss to follow-up was addressed | Methods, statistical analyses paragraph 4 |
|  |  | (*e*) Describe any sensitivity analyses | Methods, Sensitivity analyses section |
| Results | | |  |
| Participants | 13* | (a) Report numbers of individuals at each stage of study—eg numbers potentially eligible, examined for eligibility, confirmed eligible, included in the study, completing follow-up, and analysed | Sample sizes provided in Figure 1. The initial N is provided in Methods, participants section |
|  |  | (b) Give reasons for non-participation at each stage | Methods, statistical analyses paragraph 4. We have provided these reasons in total given the large number of follow-ups. |
|  |  | (c) Consider use of a flow diagram | (please see above; we have given Ns for the main waves of follow-up and have cited papers which focus on documenting and investigating follow-up across life in this cohort) |
| Descriptive data | 14* | (a) Give characteristics of study participants (eg demographic, clinical, social) and information on exposures and potential confounders | Figure 1 shows the main exposure across life (BMI) |
|  |  | (b) Indicate number of participants with missing data for each variable of interest | Figure 1; denominator for this birth cohort at initiation is 5362 |
|  |  | (c) Summarise follow-up time (eg, average and total amount) | Figure 1; follow-up is given by age |
| Outcome data | 15* | Report numbers of outcome events or summary measures over time | Figure 1 |
| Main results | 16 | (*a*) Give unadjusted estimates and, if applicable, confounder-adjusted estimates and their precision (eg, 95% confidence interval). Make clear which confounders were adjusted for and why they were included | Figures 2, 3 and 4; Figure 4 contains adjustment for polygenic score (as a potential confounder) |
|  |  | (*b*) Report category boundaries when continuous variables were categorized | n/a |
|  |  | (*c*) If relevant, consider translating estimates of relative risk into absolute risk for a meaningful time period | Supplementary information shows associations in the relative (% and SD) scales |
| Other analyses | 17 | Report other analyses done—eg analyses of subgroups and interactions, and sensitivity analyses | Results, sensitivity analyses section |
| Discussion | | |  |
| Key results | 18 | Summarise key results with reference to study objectives | Discussion, summary of findings section |
| Limitations | 19 | Discuss limitations of the study, taking into account sources of potential bias or imprecision. Discuss both direction and magnitude of any potential bias | Discussion, Strengths and Limitations section |
| Interpretation | 20 | Give a cautious overall interpretation of results considering objectives, limitations, multiplicity of analyses, results from similar studies, and other relevant evidence | Discussion, Comparison with previous studies and explanation of findings and Conclusion sections |
| Generalisability | 21 | Discuss the generalisability (external validity) of the study results | Discussion, Comparison with previous studies and explanation of findings paragraph 1 |
| Other information | | |  |
| Funding | 22 | Give the source of funding and the role of the funders for the present study and, if applicable, for the original study on which the present article is based | Funding subsection |

*Give information separately for exposed and unexposed groups.

**Note:** An Explanation and Elaboration article discusses each checklist item and gives methodological background and published examples of transparent reporting. The STROBE checklist is best used in conjunction with this article (freely available on the Web sites of PLoS Medicine at http://www.plosmedicine.org/, Annals of Internal Medicine at http://www.annals.org/, and Epidemiology at http://www.epidem.com/). Information on the STROBE Initiative is available at http://www.strobe-statement.org.
