# Supplementary figures and images for: Polygenic and socioeconomic risk for high body mass index: 69 years of follow-up across life
Source: PLoS Genet. 2022 Jul 14;18(7):e1010233. doi: 10.1371/journal.pgen.1010233 (PMC9282556; doi:10.1371/journal.pgen.1010233)

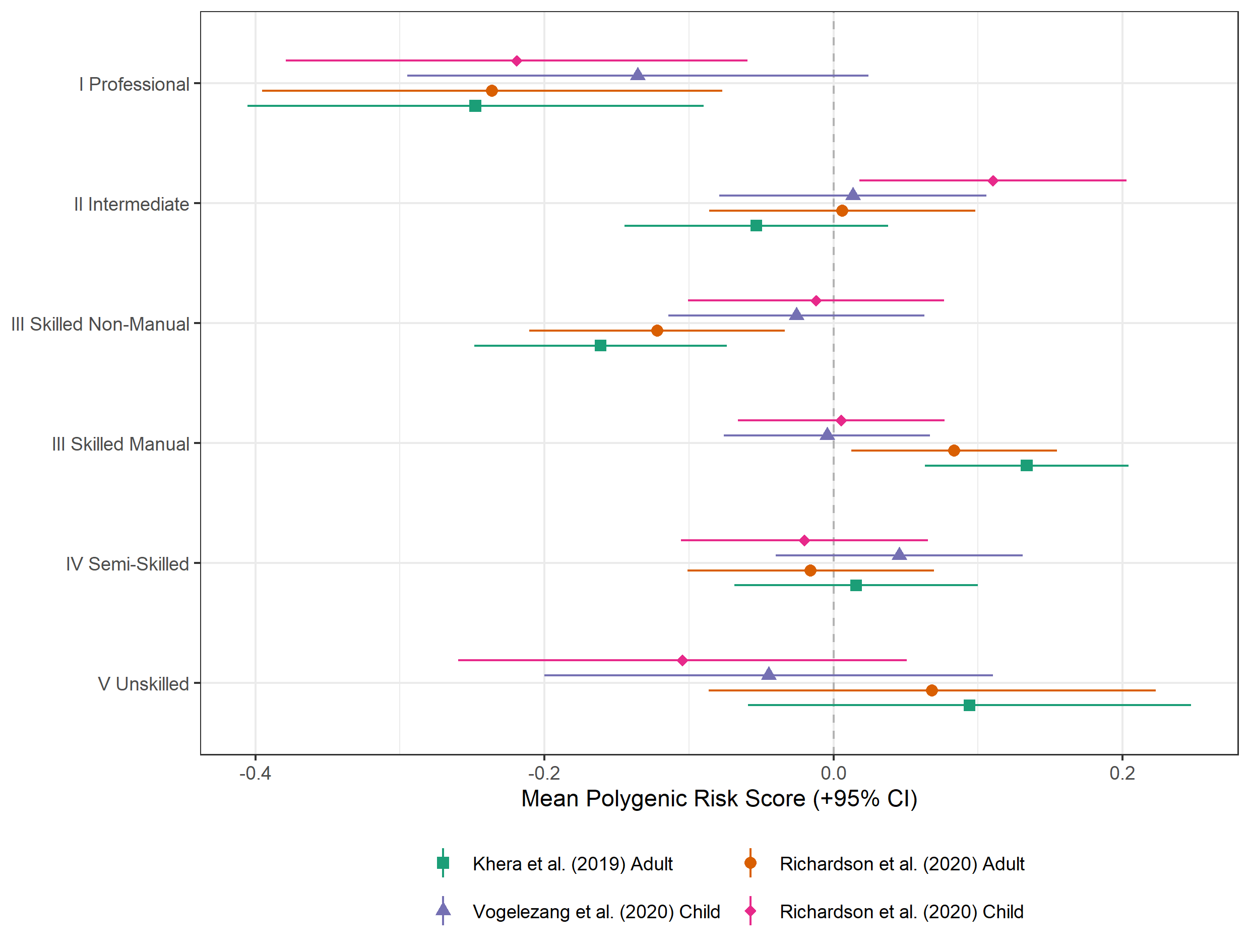


S1 Fig. Average polygenic indices by father’s occupational class at age 4 (95% confidence intervals).

Supplement: S1 Fig — (DOCX) [file pgen.1010233.s002.docx]

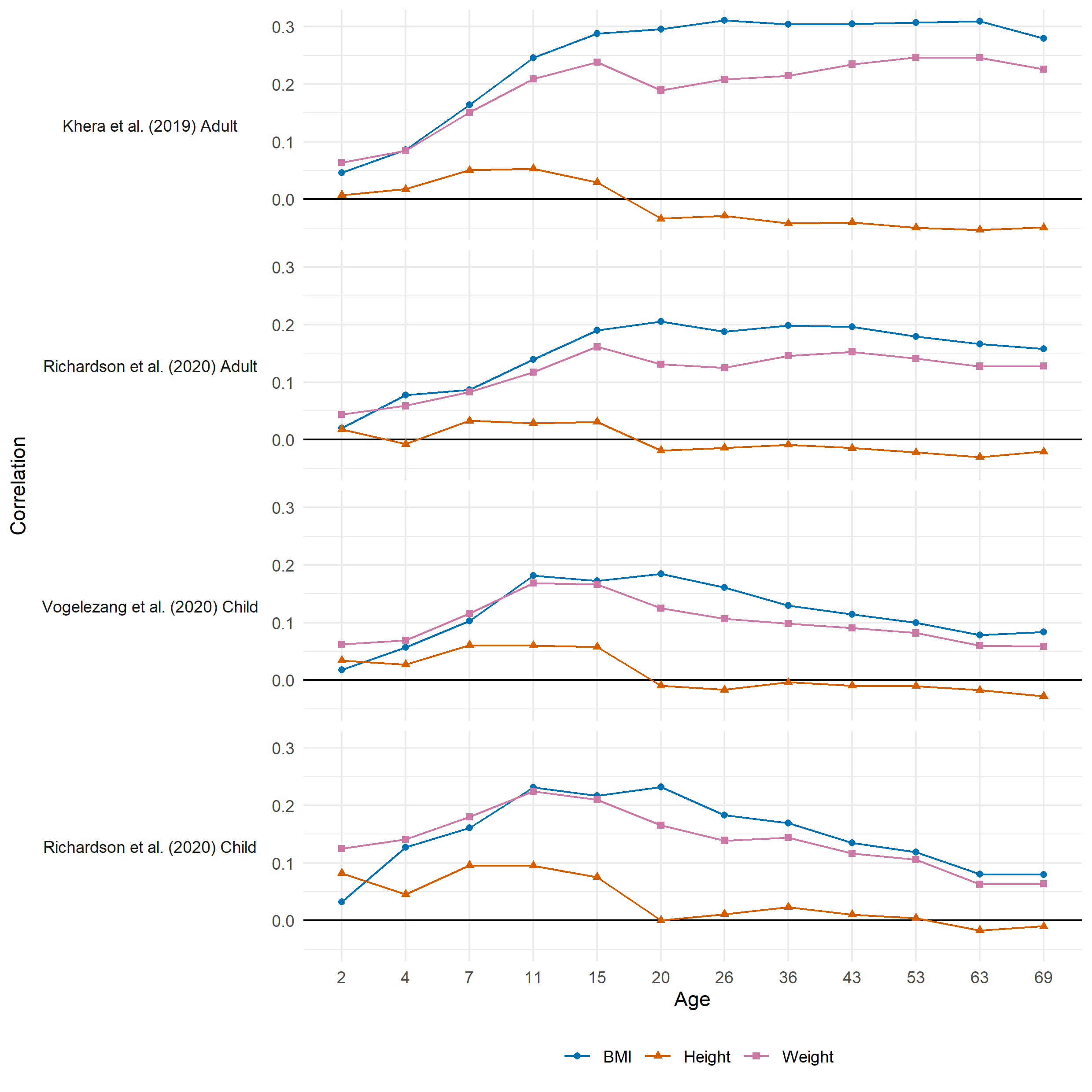


S13 Fig. Correlation between polygenic indices and BMI, height and weight by follow-up.

Supplement: S13 Fig — (DOCX) [file pgen.1010233.s014.docx]
