# Supplementary material for: Polygenic and socioeconomic risk for high body mass index: 69 years of follow-up across life
Source: PLoS Genet. 2022 Jul 14;18(7):e1010233. doi: 10.1371/journal.pgen.1010233 (PMC9282556; doi:10.1371/journal.pgen.1010233)
Supplement: S2 Fig — Standardization and percentile ranks calculated at each age at follow-up. Drawn from OLS regressions including adjustment for sex and first 10 genetic principal components, repeated for each polygenic index and age at follow up. Confidence intervals estimated using bootstrapping (500 replications, percentile method). (DOCX) [file pgen.1010233.s003.docx]

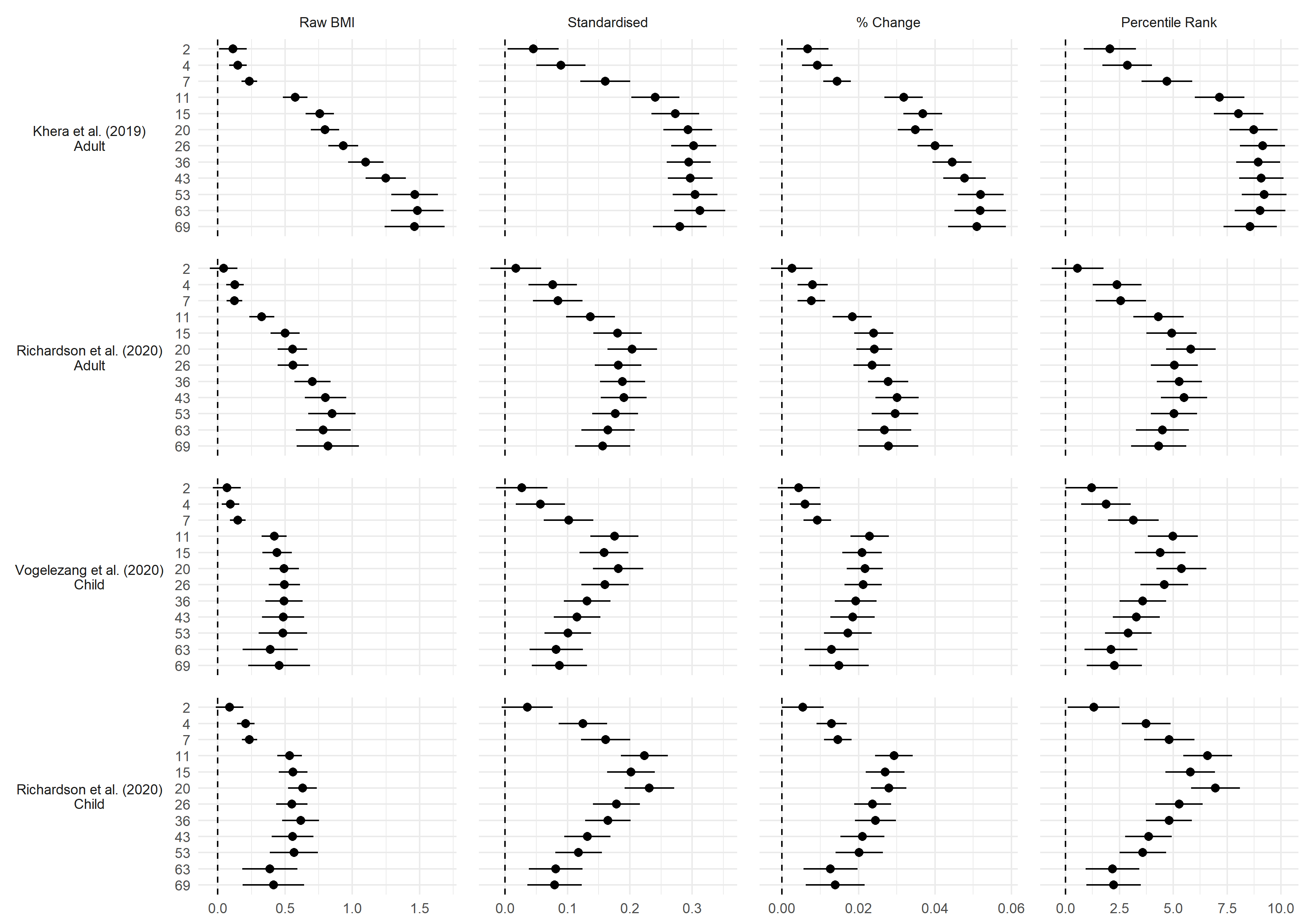


S2 Fig. Association between polygenic indices and BMI, measured as (left to right) raw scores, standardized values, logarithms, and percentile ranks. Standardization and percentile ranks calculated at each age at follow-up. Drawn from OLS regressions including adjustment for sex and first 10 genetic principal components, repeated for each polygenic risk score and age at follow up. Confidence intervals estimated using bootstrapping (500 replications, percentile method).
