# Supplementary material for: Polygenic and socioeconomic risk for high body mass index: 69 years of follow-up across life
Source: PLoS Genet. 2022 Jul 14;18(7):e1010233. doi: 10.1371/journal.pgen.1010233 (PMC9282556; doi:10.1371/journal.pgen.1010233)
Supplement: S5 Fig — Drawn from quantile regressions including adjustment for sex and first 10 genetic principal components, repeated at each follow up (panels) and decile (x-axis). (DOCX) [file pgen.1010233.s006.docx]

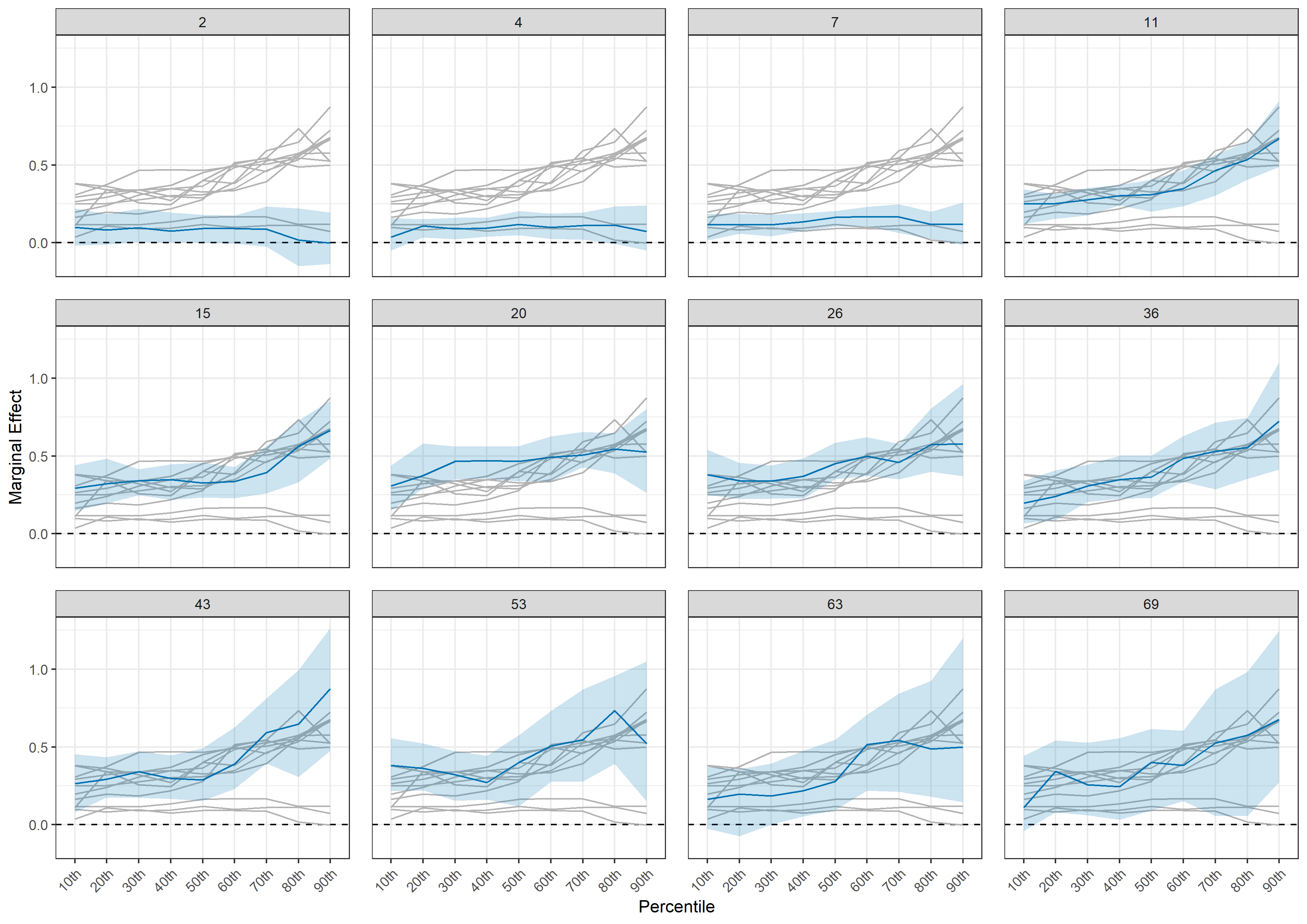


S5 Fig. Association between Vogelezang et al. (2020) polygenic index and (absolute) BMI. Drawn from quantile regressions including adjustment for sex and first 10 genetic principal components, repeated at each follow up (panels) and decile (x-axis).
