# Supplementary material for: Polygenic and socioeconomic risk for high body mass index: 69 years of follow-up across life
Source: PLoS Genet. 2022 Jul 14;18(7):e1010233. doi: 10.1371/journal.pgen.1010233 (PMC9282556; doi:10.1371/journal.pgen.1010233)
Supplement: S8 Fig — Incremental R2 compared to OLS regression model of BMI on sex and Khera et al. [4] polygenic index and first 10 genetic principal components. Multiple adjusted refers to model in which all SEP measures displayed were added simultaneously. (DOCX) [file pgen.1010233.s009.docx]

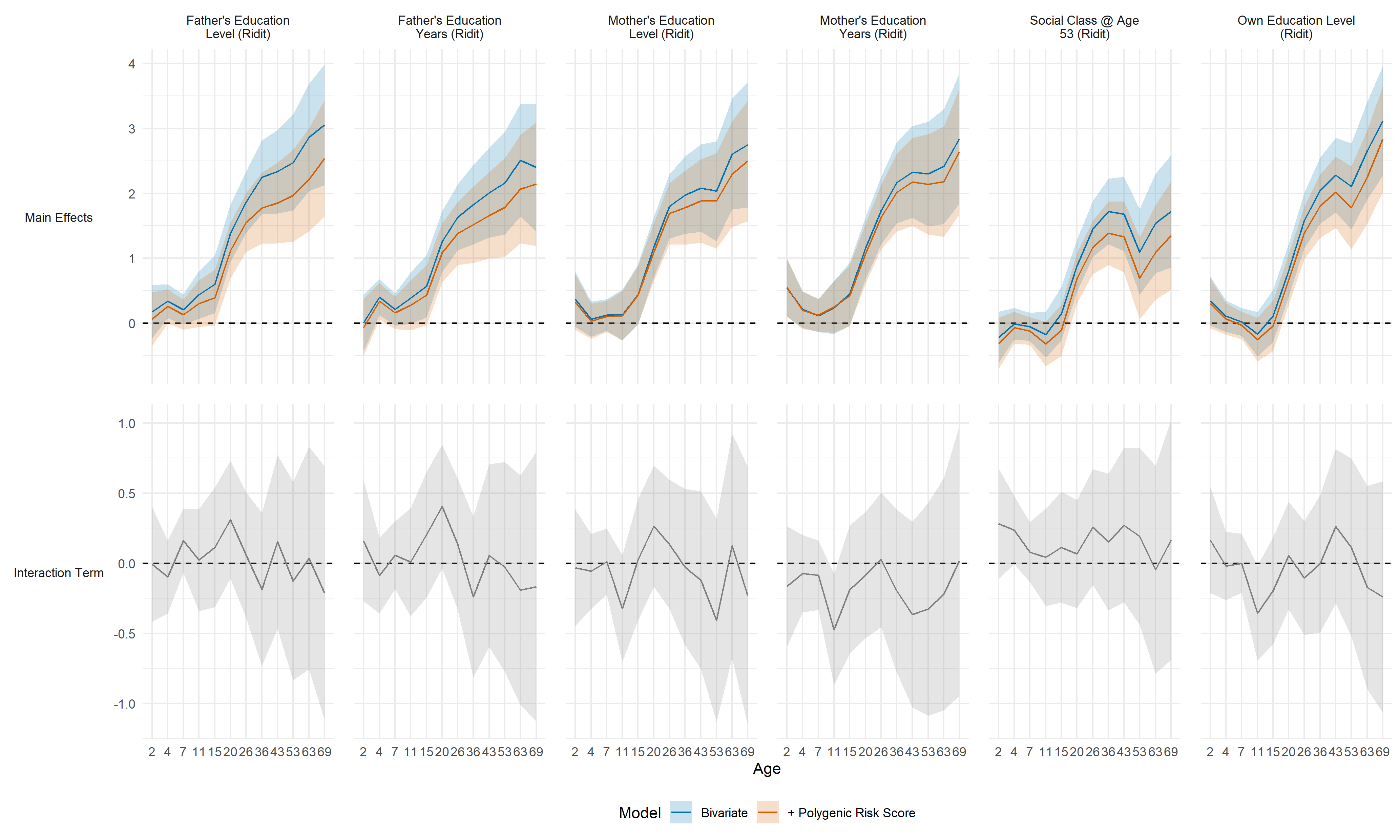


S8 Fig. Association between BMI childhood socioeconomic position and SEP and body mass index (BMI) across life, by measure of SEP. Top panel shows the kg/m^2^ difference in BMI in the lowest compared with highest socioeconomic position. Bottom panel shows coefficients for the social class x polygenic index interaction term (null line is evidence for no interaction). Results from top panel drawn from OLS regression models including adjustment for sex (blue solid line) and further adjustment for Khera et al. (2019) polygenic index and first 10 genetic principal components (orange dashed line). Results from bottom panel drawn from OLS regression models including adjusted for sex, polygenic index index (Khera et al., 2019), first ten genetic principal components and SEP.
