# Supplementary material for: Polygenic and socioeconomic risk for high body mass index: 69 years of follow-up across life
Source: PLoS Genet. 2022 Jul 14;18(7):e1010233. doi: 10.1371/journal.pgen.1010233 (PMC9282556; doi:10.1371/journal.pgen.1010233)
Supplement: S9 Fig — Null line is evidence for no interaction. Results drawn from OLS regression models repeated for each definition of childhood SEP (rows) and polygenic index (column) and including adjustment for sex, polygenic index score, first 10 genetic principal components, and SEP. (DOCX) [file pgen.1010233.s010.docx]

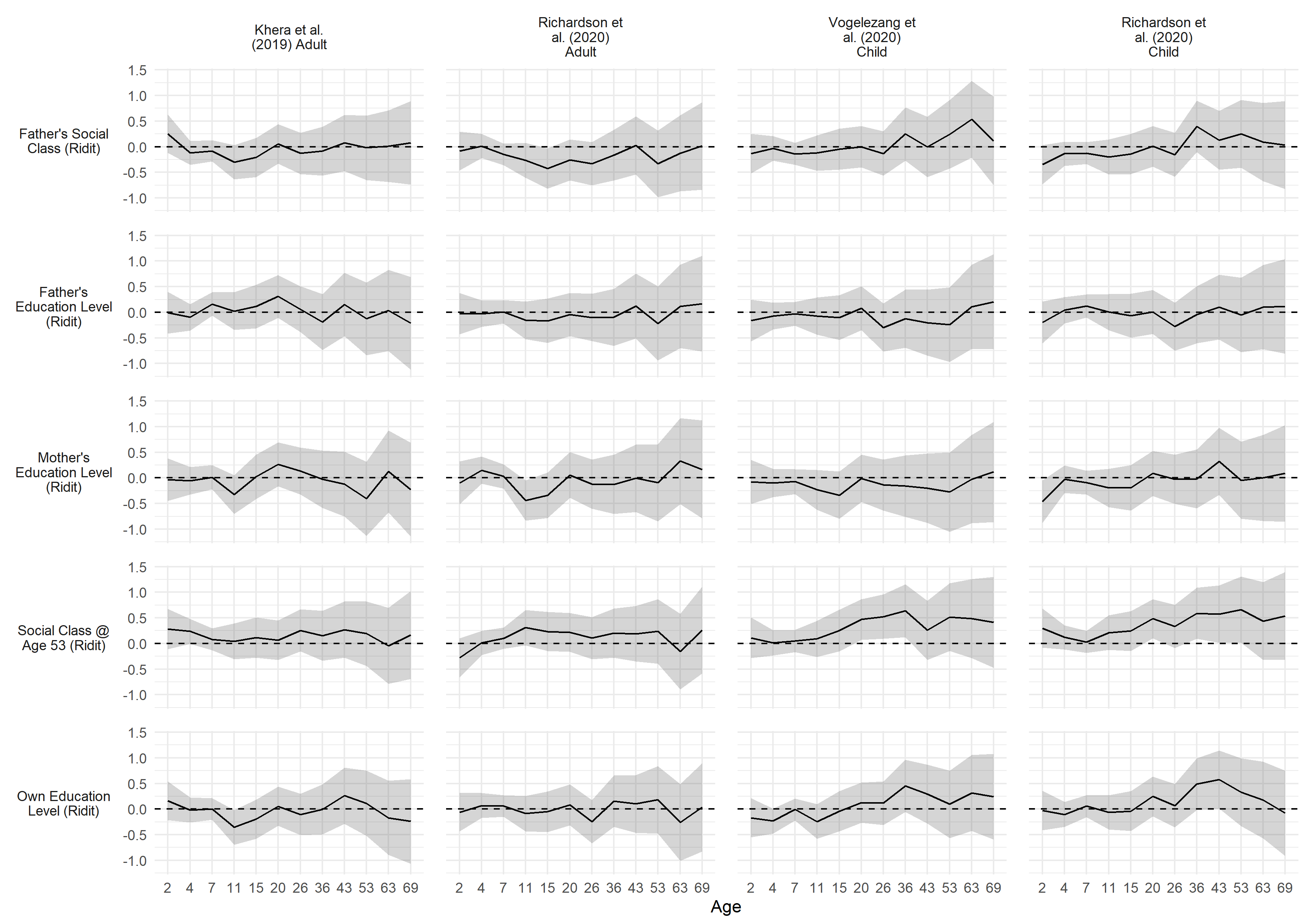


S9 Fig. Interaction effect between polygenic risk and childhood socioeconomic position and body mass index. Null line is evidence for no interaction. Results drawn from OLS regression models repeated for each definition of childhood SEP (rows) and polygenic index (column) and including adjustment for sex, polygenic index score, first 10 genetic principal components, and SEP.
