# Supplementary material for: Polygenic and socioeconomic risk for high body mass index: 69 years of follow-up across life
Source: PLoS Genet. 2022 Jul 14;18(7):e1010233. doi: 10.1371/journal.pgen.1010233 (PMC9282556; doi:10.1371/journal.pgen.1010233)
Supplement: S10 Fig — Drawn from OLS regressions including adjustment for sex and first 10 genetic principal components, repeated for each polygenic index and age at follow up. Left panel: coefficient difference in BMI per 1 SD increase in polygenic index (95% CI). Right panel: incremental R2 compared to OLS regression model of BMI on sex and first 10 genetic principal components (95% CI estimated using bootstrapping [500 replications, percentile method]). (DOCX) [file pgen.1010233.s011.docx]

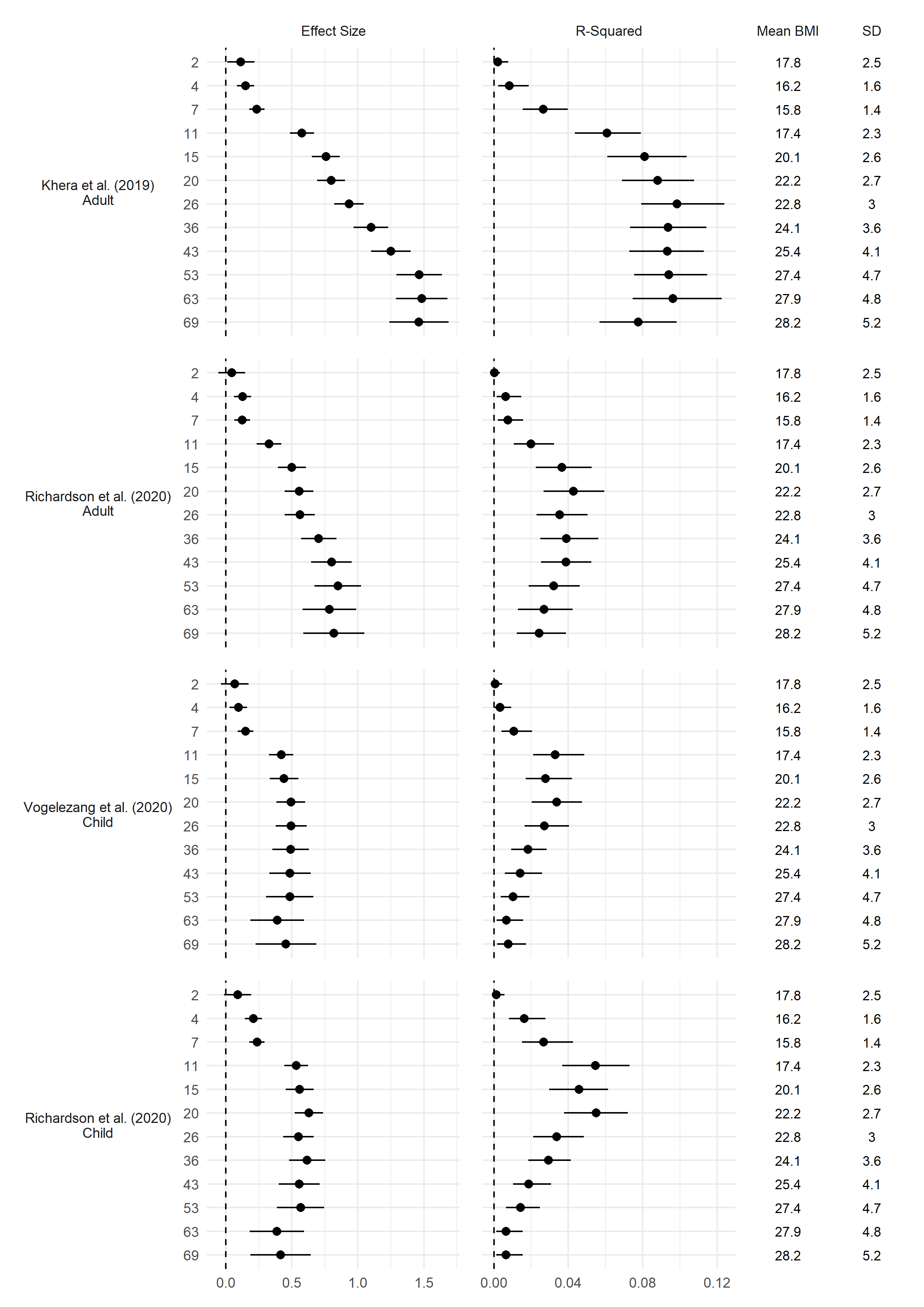


S10 Fig. Association between polygenic indices and body mass index (BMI). Drawn from OLS regressions including adjustment for sex and first 10 genetic principal components, repeated for each polygenic index and age at follow up. Left panel: coefficient difference in BMI per 1 SD increase in polygenic index (95% CI). Right panel: incremental R^2^ compared to OLS regression model of BMI on sex and first 10 genetic principal components (95% CI estimated using bootstrapping [500 replications, percentile method]).
