# Supplementary material for: Polygenic and socioeconomic risk for high body mass index: 69 years of follow-up across life
Source: PLoS Genet. 2022 Jul 14;18(7):e1010233. doi: 10.1371/journal.pgen.1010233 (PMC9282556; doi:10.1371/journal.pgen.1010233)
Supplement: S12 Fig — Standardization and percentile ranks calculated at each age of follow-up. Correction calculated by finding x such that correlation between corrected BMI (kg/mx) and height is minimized at a given age. Drawn from OLS regressions including adjustment for sex and first 10 genetic principal components and repeated for each polygenic index and age at follow up. (DOCX) [file pgen.1010233.s013.docx]

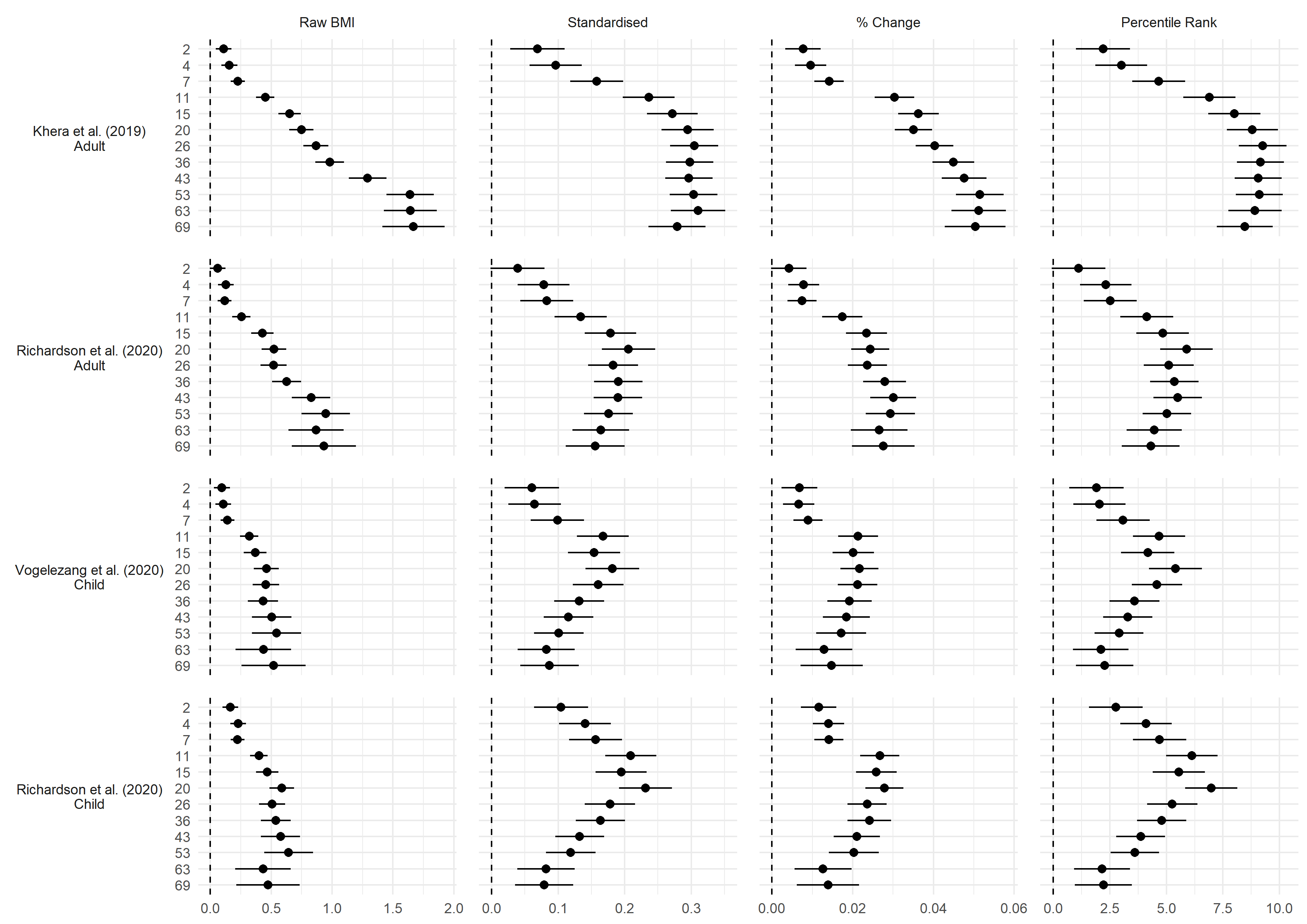


S12 Fig. Association between polygenic indices and corrected BMI, measured as (left to right) raw scores, standardized values, logarithms, and percentile ranks. Standardization and percentile ranks calculated at each age of follow-up. Correction calculated by finding x such that correlation between corrected BMI (kg/m^x^) and height is minimized at a given age. Drawn from OLS regressions including adjustment for sex and first 10 genetic principal components and repeated for each polygenic risk score and age at follow up.
