# Supplementary material for: Polygenic and socioeconomic risk for high body mass index: 69 years of follow-up across life
Source: PLoS Genet. 2022 Jul 14;18(7):e1010233. doi: 10.1371/journal.pgen.1010233 (PMC9282556; doi:10.1371/journal.pgen.1010233)
Supplement: S14 Fig — Drawn from separate regressions for each combination of PRS score (columns) and age of follow-up (rows). (DOCX) [file pgen.1010233.s015.docx]

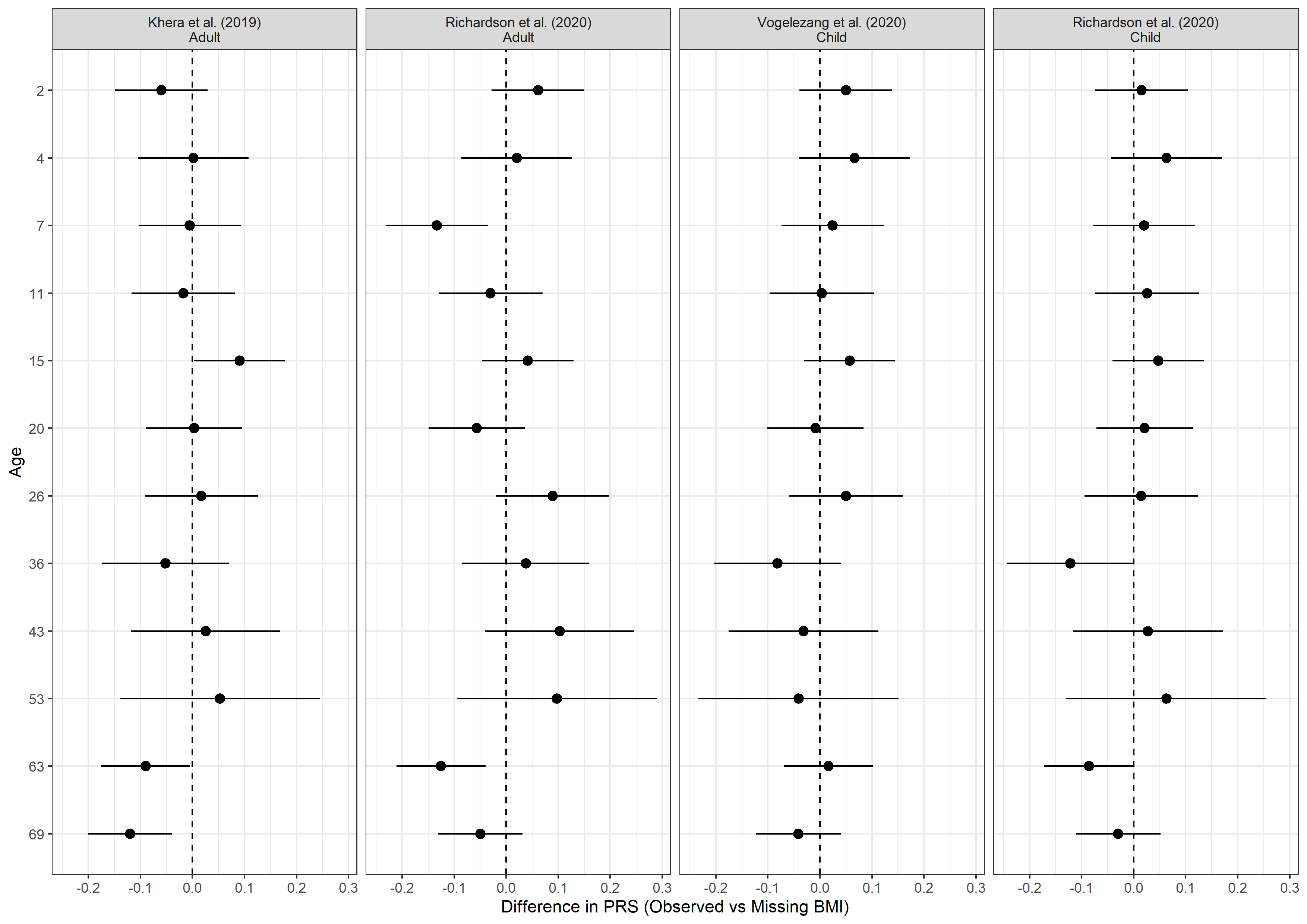


S14 Fig. Difference in average PRS scores (95% CI) by whether participant had observed or missing BMI scores at a given age. Drawn from separate regressions for each combination of PRS score (columns) and age of follow-up (rows).
