# Supplementary material for: Polygenic and socioeconomic risk for high body mass index: 69 years of follow-up across life
Source: PLoS Genet. 2022 Jul 14;18(7):e1010233. doi: 10.1371/journal.pgen.1010233 (PMC9282556; doi:10.1371/journal.pgen.1010233)
Supplement: S15 Fig — Drawn from OLS regressions including adjustment for sex and first 10 genetic principal components and repeated at each follow up using observed sample and samples of participants interviewed at each sweep following a given age (panels). (DOCX) [file pgen.1010233.s016.docx]

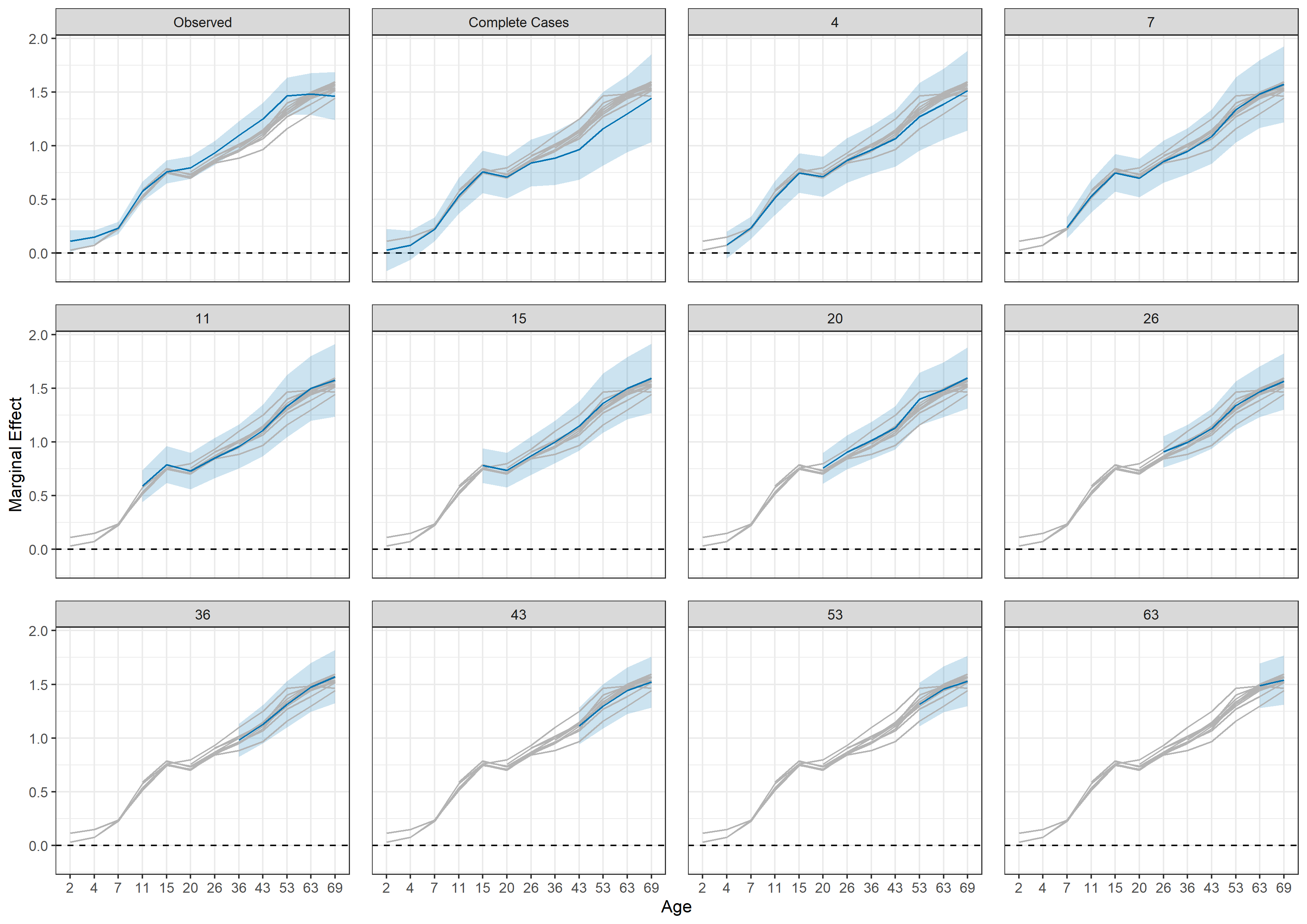


S15 Fig. Association between Khera et al. (2019) polygenic index and (absolute) BMI. Drawn from OLS regressions including adjustment for sex and first 10 genetic principal components and repeated at each follow up using observed sample and samples of participants interviewed at each sweep following a given age (panels).
