# Supplementary material for: Polygenic and socioeconomic risk for high body mass index: 69 years of follow-up across life
Source: PLoS Genet. 2022 Jul 14;18(7):e1010233. doi: 10.1371/journal.pgen.1010233 (PMC9282556; doi:10.1371/journal.pgen.1010233)
Supplement: S21 Fig — Drawn from OLS regressions compared solely adjusting for sex and first 10 principal components. Regression models repeated at each follow up using observed sample and samples of participants interviewed at each sweep following a given age (panels). Confidence intervals derived from 500 bootstrap replications using the percentile method. (DOCX) [file pgen.1010233.s022.docx]

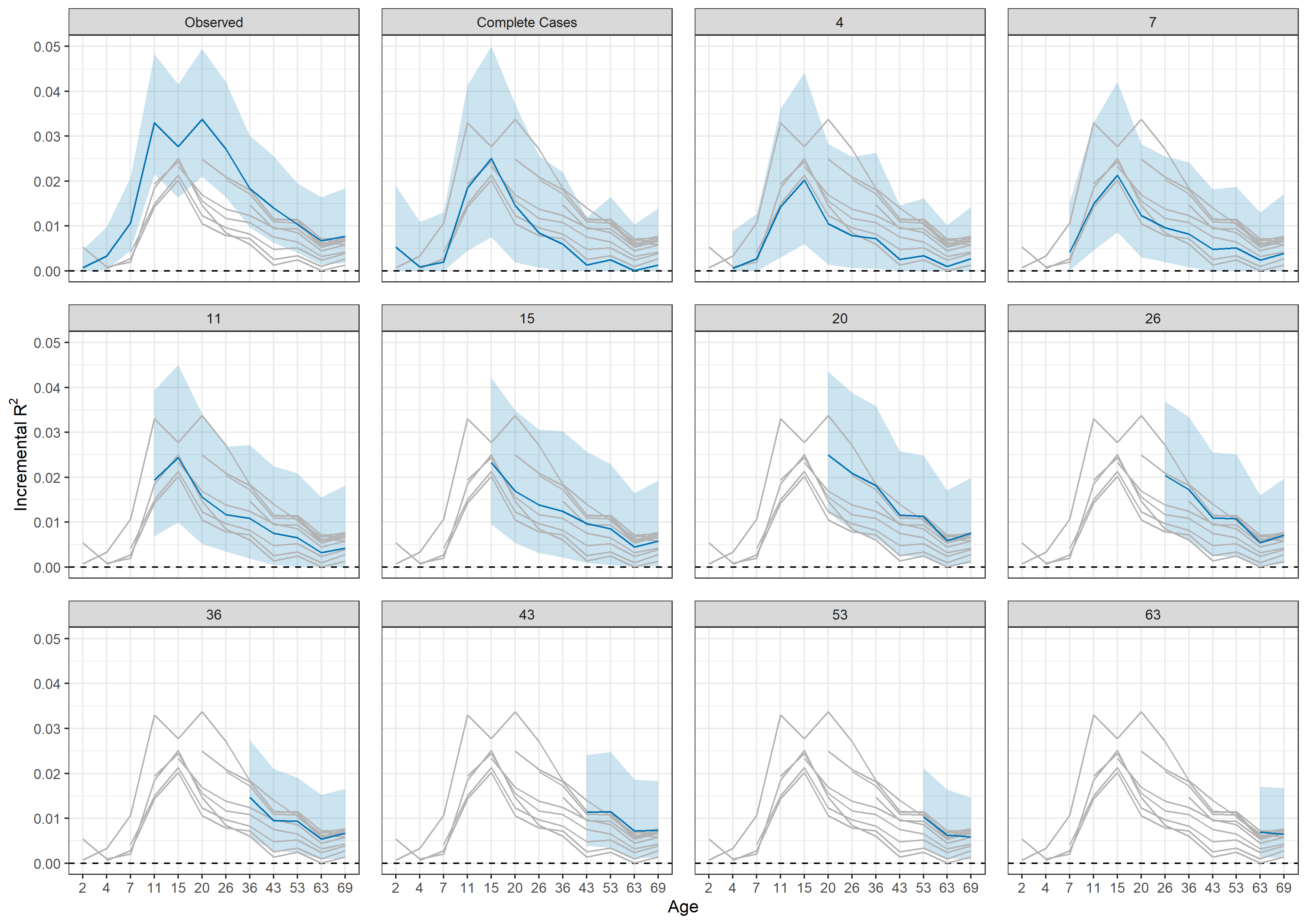


S21 Fig. Incremental proportion of variance in (absolute) BMI explained by Vogelezang et al. (2020) polygenic index. Drawn from OLS regressions compared solely adjusting for sex and first 10 principal components. Regression models repeated at each follow up using observed sample and samples of participants interviewed at each sweep following a given age (panels). Confidence intervals derived from 500 bootstrap replications using the percentile method.
