# Supplementary material for: Polygenic and socioeconomic risk for high body mass index: 69 years of follow-up across life
Source: PLoS Genet. 2022 Jul 14;18(7):e1010233. doi: 10.1371/journal.pgen.1010233 (PMC9282556; doi:10.1371/journal.pgen.1010233)
Supplement: S23 Fig — Drawn from bivariate linear regression models repeated at each age. (DOCX) [file pgen.1010233.s024.docx]

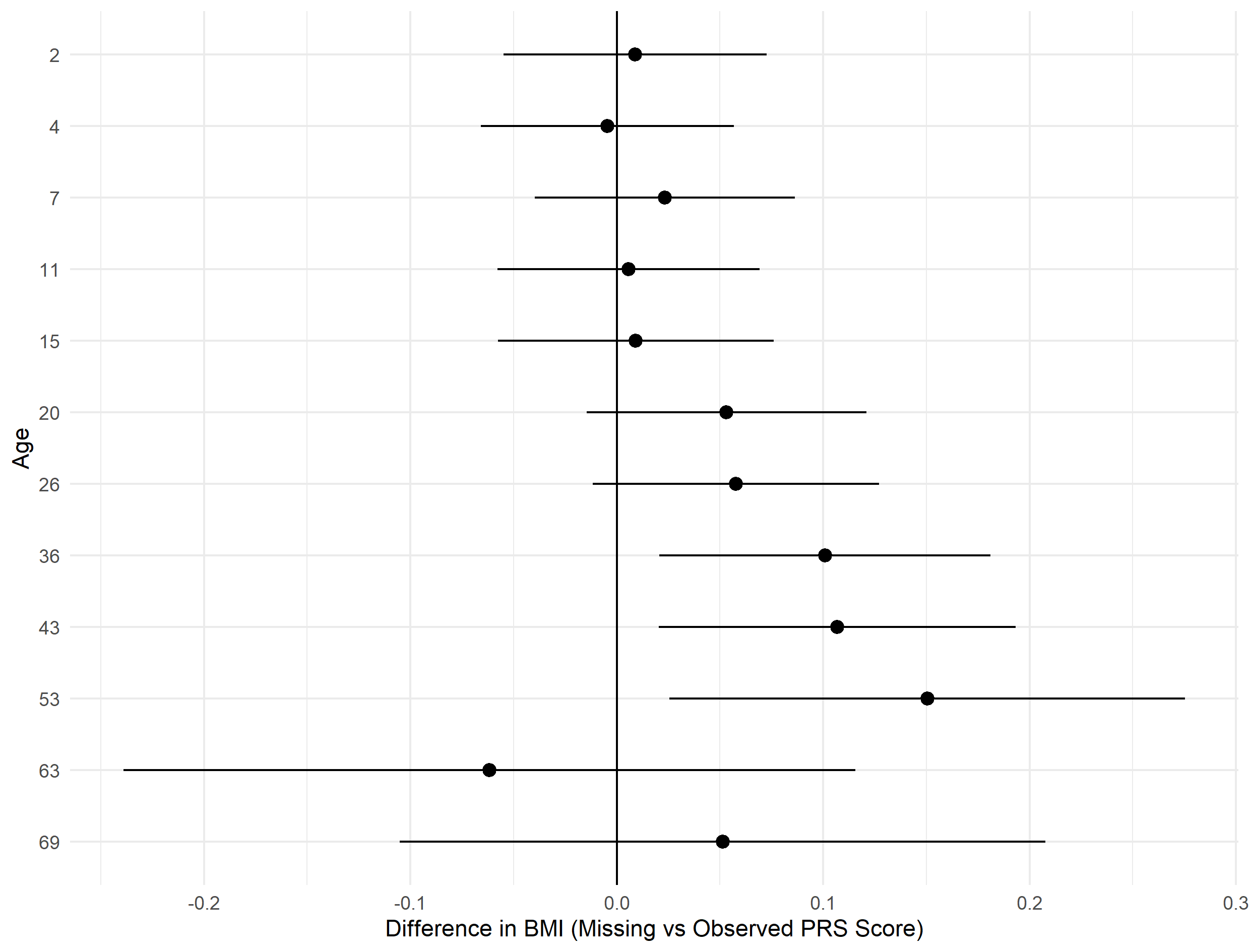


S23 Fig. Association between BMI and missing polygenic index by age at follow-up (95% confidence intervals). Drawn from bivariate linear regression models repeated at each age.
