# Supplementary material for: Polygenic and socioeconomic risk for high body mass index: 69 years of follow-up across life
Source: PLoS Genet. 2022 Jul 14;18(7):e1010233. doi: 10.1371/journal.pgen.1010233 (PMC9282556; doi:10.1371/journal.pgen.1010233)
Supplement: Attachment — Submitted filename: review.docx [file pgen.1010233.s025.docx]

Summary:

This study aims to assess the variance in BMI (at multiple life stages) explained by polygenic risk scores, and the additive/multiplicative role of socioeconomic position (SEP). This is a novel interdisciplinary research question that is answered using a rich unique data set. Few genetic epidemiology studies attempt to account for social factors, so this is to be applauded and is probably the most interesting part of the paper. My main concerns are that SEP is measured in a relatively simplistic way, and that there is not enough attention to possible confounding by genetic ancestry.

Introduction:

This section is generally well written, although it mostly focuses on genetic influences on BMI, and the only discussion of SEP is as it relates to the interaction with genetics. Need a paragraph describing research on SEP’s independent effects on BMI.

Methods:

- Given the likely rich information on SEP available for this sample, why was it measured so simplistically with just father’s occupation? This is somewhat analogous to capturing genetic risk using a single SNP. What about information on parents’ education, income, neighborhood of residence, etc? See recent paper with similar research question that captured SEP much more robustly: <https://journals.lww.com/epidem/Fulltext/2022/01000/Explaining_the_Variance_in_Cardiovascular_Disease.4.aspx>. (This paper could also be cited in the Intro/Discussion since it has a similar research question as the present study.) Or another paper with examples of other childhood SEP measures that could be incorporated, if they are available in this data set: <https://journals.plos.org/plosone/article?id=10.1371/journal.pone.0185898>.
- Even if this study was only conducted among individuals of European descent, it may be worth accounting for population stratification by adjusting for PCs for genetic ancestry. This would be especially important since you see social patterning of genetic risk, which may result from historical inequities related to individuals from different backgrounds.

Results:

- In first paragraph of Results, can you say a bit more about why it is that the polygenic indices are correlated with one another? Do they use the same SNPs, or is it known that they represent the same genes or segments of the genome?
- In first paragraph of Results, the phrase “participants from professional backgrounds” should instead refer to participants’ fathers, right?
- I would suggest moving results for one of the polygenic indices for childhood BMI (presented at the top of p. 8) to the Supplement. This is more detail than is necessary and is a lot to process, especially if the results are similar. Or if they are both important, there needs to be more of an explanation of what they each contribute.
- On p. 8, saying that the association between SEP and BMI “strengthened” is confusing. Be specific, e.g., coefficient was larger, CIs were narrower, something else?
- For the association between SEP and BMI, the very low R-squared may be due to the fact that a single measure of SEP was used. Using a more robust/sophisticated measure of SEP (as mentioned above) would be more meaningful a comparison with variance explained by polygenic scores.

Discussion:

- Need to mention limitations in generalizability, since environmental effects may differ for people in other birth cohorts, those who are not of European ancestry, those outside the UK, and for conditions other than BMI where variance explained by genetics vs SEP may be different.
- While I agree that chance does contribute to variance in BMI (middle of p. 10 Discussion), this seems to dismiss the potential for all the other aspects of SEP that were unmeasured in this study, e.g., other facets of childhood SEP, SEP in later life, etc. And again, stating that “explained variance attributable to SEP was small” fails to acknowledge that SEP was not measured robustly at all.
- These and other topics in the Discussion could also benefit from some context as provided by this paper: <https://www.ncbi.nlm.nih.gov/pmc/articles/PMC4430395/>
