# Supplementary material for: Polygenic and socioeconomic risk for high body mass index: 69 years of follow-up across life
Source: PLoS Genet. 2022 Jul 14;18(7):e1010233. doi: 10.1371/journal.pgen.1010233 (PMC9282556; doi:10.1371/journal.pgen.1010233)
Supplement: Attachment — Submitted filename: ResponseToReviewers.docx [file pgen.1010233.s026.docx]

Dear Editors

We thank the Reviewers for their careful reading of our manuscript and their positive and helpful comments.

We have revised the paper throughout, and conducted new analyses. We believe our paper is much improved and extensive (e.g., we now include 23 supplementary Figures).

Please see our point-by-point responses below,

Many thanks

Dr David Bann, Dr Liam Wright, Prof Rebecca Hardy, Dr Dylan Williams, Dr Neil Davies

Reviewer 1

Summary:

This study aims to assess the variance in BMI (at multiple life stages) explained by polygenic risk scores, and the additive/multiplicative role of socioeconomic position (SEP). This is a novel interdisciplinary research question that is answered using a rich unique data set. Few genetic epidemiology studies attempt to account for social factors, so this is to be applauded and is probably the most interesting part of the paper. My main concerns are that SEP is measured in a relatively simplistic way, and that there is not enough attention to possible confounding by genetic ancestry.

***Response: We thank the reviewer for their positive remarks. Please see below for responses to these concerns.

Introduction:

This section is generally well written, although it mostly focuses on genetic influences on BMI, and the only discussion of SEP is as it relates to the interaction with genetics. Need a paragraph describing research on SEP’s independent effects on BMI.

***Response: As suggested we have included this (Introduction paragraphs 5-6).

Methods:

-	Given the likely rich information on SEP available for this sample, why was it measured so simplistically with just father’s occupation? This is somewhat analogous to capturing genetic risk using a single SNP. What about information on parents’ education, income, neighborhood of residence, etc? See recent paper with similar research question that captured SEP much more robustly: https://journals.lww.com/epidem/Fulltext/2022/01000/Explaining_the_Variance_in_Cardiovascular_Disease.4.aspx. (This paper could also be cited in the Intro/Discussion since it has a similar research question as the present study.) Or another paper with examples of other childhood SEP measures that could be incorporated, if they are available in this data set: https://journals.plos.org/plosone/article?id=10.1371/journal.pone.0185898.

***Response:

We initially chose to focus on father’s occupation since this enables coding of childhood social class using the UK social class schema (see below response); this in turn has been strongly related to measured BMI in this cohort (eg, see <https://journals.plos.org/plosmedicine/article?id=10.1371/journal.pmed.1002214>) as well as other health and social outcomes (eg, see <https://jech.bmj.com/content/jech/early/2020/10/05/jech-2020-214423.full.pdf>).

However, in light of the reviewer’s comment we now include results for six additional SEP indicators available in this dataset—capturing maternal and paternal education, own education, and own social class. These capture two different dimensions of SEP (social class using the official UK classification system) and education. Results across all indicators of SEP are similar, with no clear evidence for interaction with polygenic indices. However, we cannot exclude the possibility that results may differ for other indicators of SEP for which we do not have available data (eg, parental disposable income or wealth). We now comment on this potential limitation (Discussion, strengths and limitations subsection) and cite Vable et al 2017 and Hamad et al (2022). We also conduct a model including all available indicators of SEP and report the r-squared (results, SEP and polygenic index in relation to BMI across life; “In a model with multiple SEP indicators added simultaneously, the maximal incremental explained variance explained was approximately 4%“).

Please also see our below response in which we caution against the comparison of r-squared values obtained for childhood SEP in comparison to polygenic indices.

-	Even if this study was only conducted among individuals of European descent, it may be worth accounting for population stratification by adjusting for PCs for genetic ancestry. This would be especially important since you see social patterning of genetic risk, which may result from historical inequities related to individuals from different backgrounds.

***Response: As suggested we now include adjustment for PCs for genetic ancestry (see Figure 1). We agree that adjusting for this is justified a-priori and thank the reviewer for their suggestion; our results however do not change after this adjustment (eg, see Figures 1-3).

Results:

-	In first paragraph of Results, can you say a bit more about why it is that the polygenic indices are correlated with one another? Do they use the same SNPs, or is it known that they represent the same genes or segments of the genome?

***Response: We believe that this correlation is likely due to some combination of the same SNP coverage and linkage disequilibrium (that is, SNPs which tend to co-occur together). As suggested we now discuss this possibility in the Discussion (Discussion, Comparison with previous studies and explanation of findings paragraph 3). We would be happy to expand upon this (eg, in the supplement) if requested.

-	In first paragraph of Results, the phrase “participants from professional backgrounds” should instead refer to participants’ fathers, right?

***Response: We have amended the wording to refer to childhood social class; in the UK social science literature this is typically indicated by father’s occupation—this schema was used as the official UK social class schema in official statistics (eg, for applied examples of this in epidemiological research see <https://journals.plos.org/plosmedicine/article?id=10.1371/journal.pmed.1002214> and <https://www.sciencedirect.com/science/article/pii/S2468266718300458> and <https://jech.bmj.com/content/66/6/512.short>).

-	I would suggest moving results for one of the polygenic indices for childhood BMI (presented at the top of p. 8) to the Supplement. This is more detail than is necessary and is a lot to process, especially if the results are similar. Or if they are both important, there needs to be more of an explanation of what they each contribute.

***Response: We thank the reviewer for this suggestion. As suggested we have moved results of one polygenic index to the supplement (Richardson et al’s childhood BMI polygenic index). We also focus main analyses on the results of one polygenic index (Khera et al in Figs 1-4); others are moved to a separate Figure (Fig 5) to aid comparison (and the supplement). In fig 5, we additionally include analysis using a polygenic index for adulthood BMI (restricted to significant hits, consistent with the childhood BMI scores); this provides an alternative comparison (conclusions do not differ).

-	On p. 8, saying that the association between SEP and BMI “strengthened” is confusing. Be specific, e.g., coefficient was larger, CIs were narrower, something else?

***Response: We have amended the text to be more specific (ie, that coefficients were larger).

-	For the association between SEP and BMI, the very low R-squared may be due to the fact that a single measure of SEP was used. Using a more robust/sophisticated measure of SEP (as mentioned above) would be more meaningful a comparison with variance explained by polygenic scores.

***Response: As suggested we now note the explained variance attributable to all SEP indicators used (Results, polygenic indices and SEP section). We further caution against comparison of explained variance attributable to genetic factors and SEP indicators in the Discussion (Discussion, Comparison with previous studies and explanation of findings paragraph 4).

Discussion:

-	Need to mention limitations in generalizability, since environmental effects may differ for people in other birth cohorts, those who are not of European ancestry, those outside the UK, and for conditions other than BMI where variance explained by genetics vs SEP may be different.

***Response: As suggested we now include discussion of generalizability (Discussion, Comparison with previous studies and explanation of findings paragraph 1)

-	While I agree that chance does contribute to variance in BMI (middle of p. 10 Discussion), this seems to dismiss the potential for all the other aspects of SEP that were unmeasured in this study, e.g., other facets of childhood SEP, SEP in later life, etc. And again, stating that “explained variance attributable to SEP was small” fails to acknowledge that SEP was not measured robustly at all.

***Response: We have amended this sentence and removed the reference to explained variance for SEP being small since this may be misleading given its measurement (Discussion, Comparison with previous studies and explanation of findings paragraph 3).

-	These and other topics in the Discussion could also benefit from some context as provided by this paper: https://www.ncbi.nlm.nih.gov/pmc/articles/PMC4430395/

***Response: We thank the reviewer for the suggestion and hope our revised Discussion is acceptable.

Reviewer 2:

This paper analyses several somewhat understudied topics related to the genetics of BMI using British longitudinal data. There are a many topics covered in this paper (changing associations during the life course, SES*G interactions and skewness) making the paper somewhat difficult to read. I have only a couple of comments the authors may want to consider.

***Response: We thank the reviewer for their comments. Per Reviewer 1’s suggestion, we have moved some results to the supplement. We hope this and other edits make the paper easier to read; we aimed to be ambitious in the scope of our paper rather than producing multiple papers of more limited scope.

Some of the references citied are a bit strange. For example, Mackenbach 2020 does not give evidence on the associations between SEP and genetic factors. Also the evidence for G*SEP (references 16-18) is somewhat mixed. Generally, I think that the references should be discussed more critically.

***Response: We have updated our discussion of the previous literature to provide more detail and be more exacting on what was previously found (please see our response to reviewer 1 above).

Interestingly, the genetic variation of BMI seems to follow closely the same age pattern as found previously in large scale twin studies (Silventoinen K et al. Am J Clin Nutr 2016 and Silventoinen K et al. Am J Clin Nutr 2017), i.e., having the peak in early adulthood and then declining. I am wondering whether it would be useful to make more systematic comparison to these twin studies to show that these two methods produce very consistent results.

***Response: We thank the reviewer for this interesting comment. We now include a more direct comparison of twin study findings, e.g., see the Discussion, Comparison with previous studies and explanation of findings, paragraph 1). We believe that future work in larger cohorts could use alternative methods to aid heritability comparisons with twin studies. For example, testing narrow sense heritability estimates using GCTA by age—we attempted this in response to this comment, yet found insufficient statistical power in this sample (estimates which were very imprecise eg, of 0.49 (SE 0.36) at 20-years and 0.64 (SE 0.38) at 60-64 years).

Minor comments

Page 3: the first sentence: The prevalence of BMI has probably not change (mean of BMI has increased of prevalence of overweight/obesity increased).

***Response: We have amended this sentence to note that increases in mean BMI and its variance also correspond to increases in overweight and obesity prevalence (Introduction paragraph 4).

Reviewer #3:

Dear authors,

Thank you very much for the opportunity to review your research.

This manuscript reads very well and and really enjoyed reading it

***Response: We thank the reviewer for this positive comment.

However, I do not think that this is anywhere ready for submission. The data presented and the interpretation lacks robustness and in many ways looks preliminary.

***Response: Please see our responses to Reviewers 1 and 2 above; we have revised our manuscript and would be happy to do so again if there are specific suggestions.

We believe our manuscript is not preliminary. Indeed, we build substantially on more limited previous analysis of this cohort sample which have made considerable unique contributions to the literature (eg, Hardy et al used single variants only, did not analyse SEP or quantile regression, and has been cited 275 times; please see <https://pubmed.ncbi.nlm.nih.gov/19880856/>). We also include a completed STROBE checklist and extensive results (23 Supplementary Figures).

For example I am surprise that such a great team does not provide the necessary descriptive of the population to allow the reader to ascertain the selection factors in the cohort they studied. Childhood SEP being an important factors of not participation, this is very important to inform (in addition to many other possible confounders or selection factors).

***Response: We now include additional information on the sample and loss to follow-up in this cohort (Methods, Participants). We cited papers which have extensively examined patterns of loss to follow-up empirically. We include analyses to explicitly consider the extent to which our findings may be biased by selection (see sensitivity analyses section, copied below, and discuss the potential for bias in our Discussion (Limitations):

Methods:

“Our main analyses were conducted using those with observed BMI data at each age. Due to loss to follow-up, sample sizes differ across age. To explore whether this influenced our results, we 1) investigated whether polygenic indices were related to whether the participant had observed BMI at a given age; and 2) repeated analyses in samples with valid data for all follow-ups from a given age up to age 69, iterating across follow-ups (e.g., those followed from age 2 had complete case data at all timepoints, while those followed-up from 53 years had valid data from 53-69 years).”

Results:

“Polygenic indices were related to missing BMI data at some ages (Fig S14). Notably, non-missing BMI data at ages 63 and 69 was related to lower than average Khera et al. polygenic indices. Investigation of associations between polygenic indices and BMI using samples of the same participants across time showed broadly similar results as the main analysis (Figs S15-S22). However, there was some evidence that the plateauing of effect sizes in the Khera et al. and Richardson et al. (adult) index-BMI associations from 53 to 69 years was an artefact of differences in the samples at each age—when using the same sample across this age span, effect sizes were slightly higher at ages 63 and 69 (Figs S15-16).”

An other example is the lack of justification of the use of Childhood SEP in this study. Why no adult SEP indicator used?

***Response: Please see the response to Reviewer 1 above in which we expand on the SEP indicators used. We focused on childhood SEP to limit bias due to reverse causality (see Methods, Socioeconomic position section). Findings were similar when using multiple alternative SEP indicators.

An other example suggesting that the manuscript is still at its preliminary stage is to suggest increase BMI with age not considering that the average BMI in childhood and adulthood are not comparable (in terms of absolute value).

***Response: We used multiple methods to aid comparability of childhood and adult BMI including the use of relative differences in BMI (e.g. ranks) and the use of a corrected BMI variable (kg/m^x^) where x was chosen at each age such that BMI was not correlated with height. Please see below for the text used in the manuscript. We believe that we have gone further than previous work to address this topic, are not aware of additional tools to aid comparability; however, we welcome further suggestions.

Statistical analysis (paragraph 1)

“We investigated associations on the absolute (kg/m2) and relative (percentage, percentile rank and standardized score) scales separately, since each may be informative; the absolute scale may aid comparability of effect sizes across adulthood as a 1 unit increase in BMI may have equivalent health risk; the relative scale may aid comparability across childhood and adulthood given sizable differences in mean BMI and its variation across age.”

Statistical analysis (sensitivity analyses subsection)

“We then investigated if the associations were driven by weight and/or height – BMI (kg/m2) is a ratio measure and thus could reflect associations with height, particularly at younger ages. To account for this, we estimated separate associations with weight, height, and BMI; we also calculated a corrected weight-for-height index, dividing weight (kg) by height raised to a power that reduced the correlation between height and the index to zero at each age. “

This is really a pity as the data look fascinating. Considering the importance of such health information and the ethical responsibility to reduce speculation in social and genetic inheritance of leaving with obesity, I can only recommend to strengthen your data by following the STROBE recommendations as instructed by the journal.

***Response: We have included a STROBE checklist in which all sections are completed as supplementary material.
